# Electrically Conductive Bulk Composites through a Contact-Connected Aggregate

**DOI:** 10.1371/journal.pone.0082260

**Published:** 2013-12-09

**Authors:** Ahsan I. Nawroj, John P. Swensen, Aaron M. Dollar

**Affiliations:** Mechanical Engineering and Materials Science, School of Engineering and Applied Science, Yale University, New Haven, Connecticut, United States of America; Queen's University at Kingston, Canada

## Abstract

This paper introduces a concept that allows the creation of low-resistance composites using a network of compliant conductive aggregate units, connected through contact, embedded within the composite. Due to the straight-forward fabrication method of the aggregate, conductive composites can be created in nearly arbitrary shapes and sizes, with a lower bound near the length scale of the conductive cell used in the aggregate. The described instantiation involves aggregate cells that are approximately spherical copper coils-of-coils within a polymeric matrix, but the concept can be implemented with a wide range of conductor elements, cell geometries, and matrix materials due to its lack of reliance on specific material chemistries. The aggregate cell network provides a conductive pathway that can have orders of magnitude lower resistance than that of the matrix material - from 10^12^ ohm-cm (approx.) for pure silicone rubber to as low as 1 ohm-cm for the silicone/copper composite at room temperature for the presented example. After describing the basic concept and key factors involved in its success, three methods of implementing the aggregate into a matrix are then addressed – unjammed packing, jammed packing, and pre-stressed jammed packing – with an analysis of the tradeoffs between increased stiffness and improved resistivity.

## Introduction

**Figure 1:**
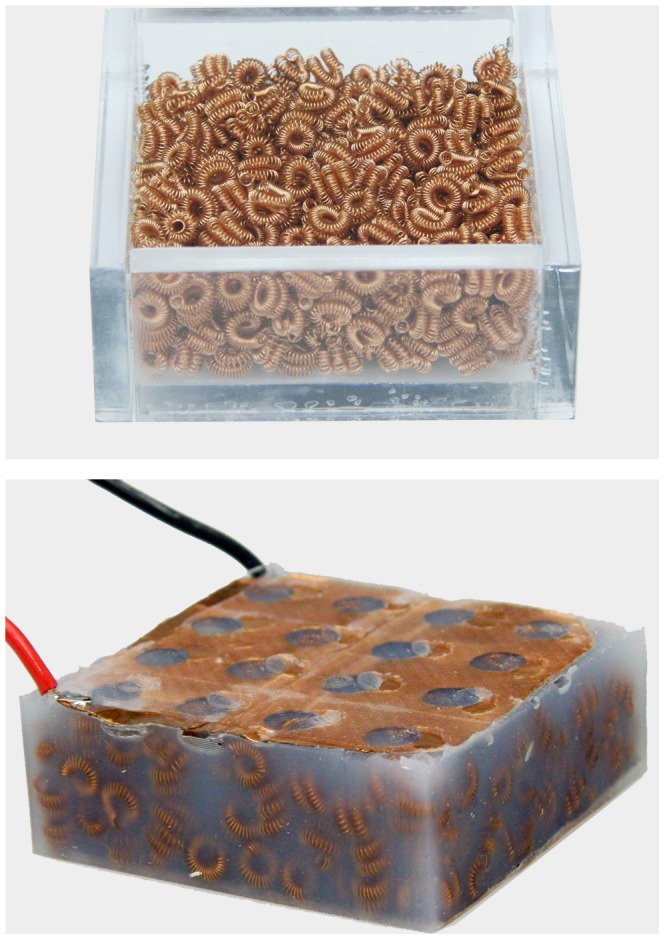
Photograph of a sample cell packing (top) and composite (bottom). The cell-packing forms the internal skeleton of the composite. Pouring and curing a polymeric mixture (Silicone 10 used here) around this skeleton creates a composite sample.

There are a large number of reasons why it might be desirable to enable a component or structure to be electrically conductive without limiting the choices of materials to metals or the small number of conductive non-metals. In doing so, material considerations including mechanical properties such as strength, density, or modulus, as well as other factors such as material cost, compatibility, and availability can be prioritized separately from the electrical properties. Along these lines, this paper describes a concept in which any number of materials can be composited with a network of simple conductive cells in order to produce highly conductive structures of nearly arbitrary size or shape. While these cells might be made from any conductive material and in a wide range of geometries, we demonstrate the concept with copper cells that are fabricated into coils-of-coils, which have a relatively low stiffness and low volumetric density while allowing for a large number of contacts with surrounding cells (Figure 1). A key feature of the success of the concept relates to inducing a sufficient level of force between cells in order to minimize the contact resistance, without which the resistivity of the structure will be much higher (although still possibly acceptable, depending on the application). Due to the fact that the concept does not rely on specific material chemistries, it can be implemented in nearly any matrix material, provided that the aggregate can be embedded within the solid-form structure during fabrication.

We believe that one promising implementation of the proposed work is in the area of conductive polymers, most of which are being developed for applications that require a low-stiffness material. Related work is in two primary areas: intrinsically conductive polymers and conductive polymer composites. The former relies on the polymerization chemistry to create conductive chains throughout the material, creating either metallic conductivity or acting as semiconductors. Research has been done on Polyaniline to decrease its resistivity from 10^10^ ohm-cm to 1 ohm-cm [1]. Halogenization of thin film conductive polymers with iodine vapor has been reported to reduce resistivity in intrinsically conductive polymers from 10^4^ ohm-cm to as low as 10^−3^ ohm-cm, though their lifetime is limited after removal from the concentrated vapors [2] [3].

Most relevant to the proposed concept, conductive polymer composites merge the useful conductive properties of a conductive aggregate with the desirable properties of polymer matrix (including elasticity, tensile strength, corrosion resistance, low-cost, and accessibility). Carbon black is likely the most widely used aggregate material for conductive polymer composites [4], with composite resistivities as low as 10^2^ ohm-cm. Another procedure is metal-polymer compaction, fusing conductive and non-conductive powders such as silver/Bakelite [5], copper/PMMA [6], and silver/epoxy [8], with composite resistivities as low as 10^1^ ohm-cm. In terms of non-polymeric composite materials that are conductive, significant experimental efforts have been conducted for concrete-based resistive composites for ohmic heating of bridges and highways [7], [8]. Other research has been done on rigid glass fiber reinforced plastic (GRFP) infused with carbon black with the mass fraction near the percolation threshold for fracture and damage sensing [9], [10]. An applications-centric review of conductive materials based on carbon additive, both rigid and flexible, is found in [11].

The proposed work differs from previous efforts in a number of ways. As stated earlier, it does not rely on any particular material chemistry, as opposed to the work on intrinsically-conductive polymers. While it is most similar to the technique of the metal-polymer compaction techniques [6]–[8], it is not strictly a powder-based process and the density of the conductive aggregate is an order of magnitude lower.

The remaining sections of this paper are organized as follows. First, we describe simulations performed using model cell geometry and increasing numbers of cells in a jammed packing. The simulations provide insight into an appropriate number of cells to create viable packings providing connectivity throughout the composite structure. Next we detail the concept of a cell, our implementation of different types of cell packings, varying packing fractions in order to improve conductivity at the expense of composite stiffness. We go on to summarize experimental testing of these alternative implementations and characterize their performance and additionally explore the effect of applied loads on the resistance of the composites.

### Simulations of Cell Packings

In order to better understand some of the key factors affecting the performance of the proposed composites, we created a simulation environment based on jammed packings of cells, which is closely related to the proposed fabrication process. Our simulations were designed to assess the effects of boundary conditions and packing variability on the bulk resistance of the conductive composite. Properties to be investigated included the variability between samples (packings) as a function of number of cells and as a function of the applied stress on the sample. In order to gain insight into these effects while keeping the simulation complexity manageable, we examined spherical cells with the mechanical and electrical properties of copper to explore the results of packing and jamming on the bulk conductivity of the aggregate ensemble and the composite. Using the approximate dimensions of the copper cells and packing mold to be used in the physical experiments (section III.A.), cells are packed together using a physics simulation of elastic spherical particles with viscous damping, as described in [12]. Each cell is assumed to be an elastic particle with appropriate coefficients of stiffness and damping. Five sides of the rectangular bounding box are fixed and the upper boundary is slowly moved until jammed packing is achieved with one of four different specified pressures at the upper and lower boundaries. The packing simulations are continued until the packing pressure is satisfied and the sum of the kinetic energies for all cells has settled sufficiently. Subsequently, the resistive network is analyzed as in [13].

The physics simulation assumed each cell could be characterized by a spherical stiffness under compression and a viscous damping with the surrounding region. The orientation of each cell was irrelevant as we assumed that the resistance through each cell was zero and the cell-to-cell contact resistance was a function of the intercellular forces. Two plate terminals were connected to all the cells on the top surface of the packing and on the bottom surface of the packing. From a macroscopic view of the entire structure, it was expected that the packed structure should begin to look like a bulk conductor, where the degree of homogeneity is related to the degree of cell connectivity and cell-to-cell forces.

**Figure 2:**
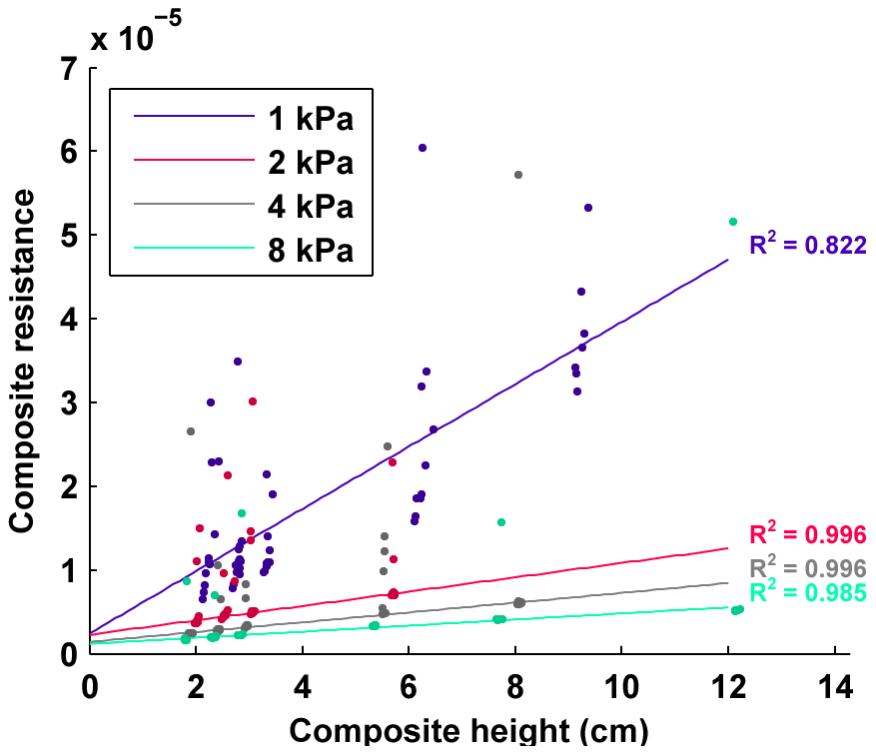
Simulation result of packing resistance vs. height of packing. Simulations show that the resistance of the packing of cells increased linearly with length (i.e. the number of cells is increasing with a fixed cross-sectional area). Also, the resistance decreases as the applied pressure increases.



**Figure 3:**
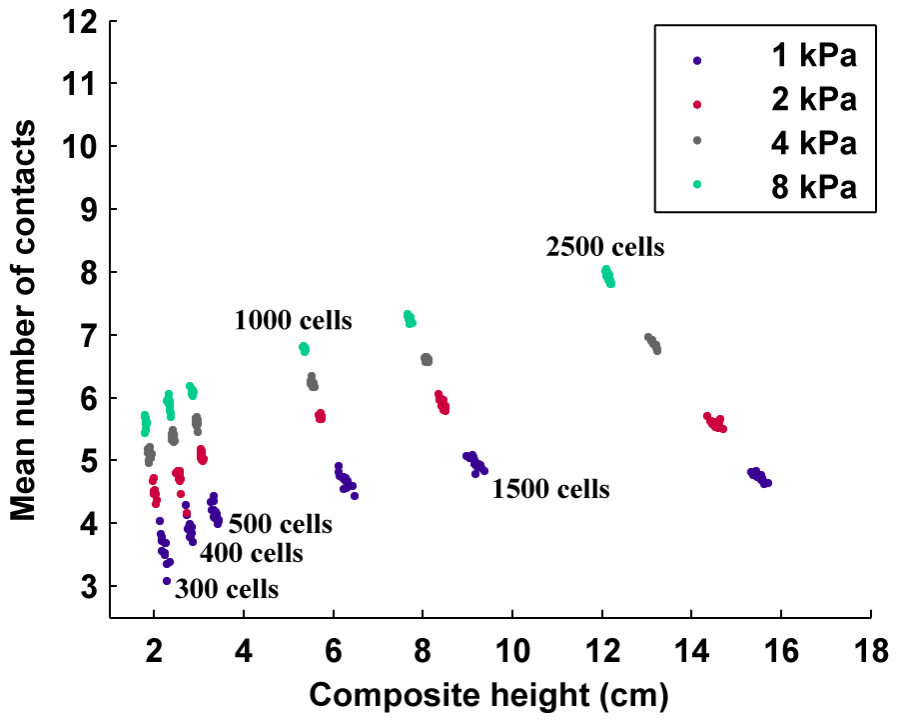
Simulation result of the mean number of neighbor-neighbor contacts per cell with increasing number of cells in the packing. The degree of parallelization in the network of packed cells is highly dependent on the cell-to-cell contacts with its neighbors. As the number of cells increases, and as the pressure on the packing increases, the mean number of contacts also increase, thus maximizing connectivity.

In Figure 2, we see how the resistance of the packing increases as the number of cells increases, for four different packing pressures. For each pressure, the increase in resistance is linear with length, as evidenced by fitting a line with high R-value. Each of these fit lines takes the form

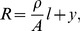
where *R* is the composite resistance, *y* represents the resistive offset due to the plate terminals, *l* is the length of the packing, *A* is the cross sectional area of the packing, and *ρ* is the resistivity. A good line fit with a constant slope is indicative of constant resistivity, which is the expected behavior of a bulk conductor with constant cross sectional area. Figure 3 also shows that there is a clear lower bound on the resistance of the packed composite at each pressure. In all of the simulations, except for the very low pressure case of 1 kPa, all of the outliers lie above this line fitting the data points for a specific pressure, indicating that their increased resistance was due to either occasional degenerate packing configurations or insufficient packing time. These outliers constitute approximately 8% of all of the simulation trials. Furthermore, Figure 3 shows that as the length of the conductor increases, the average number of contacts, or average kissing number (or contact number), for each cell begins to asymptote. The primary factor in the low average number of contacts at low cell counts is the effect of a reduced number of contacts for cells on the boundaries. The theoretical maximum kissing number (mean number of contacts) for spherical close packings is k = 12 [14], however random packings with finite settling time are expected to have far less than the optimal.

In terms of the work described here, these results show a number of things. First, the variability between packings with greater than 300 cells (approximately 2 cm in packing height) is very low, indicating that experimental testing on just a few number of samples should give results that are reflective of the norm. This is also echoed by the fact that the resistance varies linearly with number of cells even at the low end of sample height. Next, the resistance varies strongly with applied pressure, but the effect becomes smaller after an initial large jump (before 2 kPa in this simulation). Finally, the mean number of contacts is more of a function of applied stress than number of cells, generally reaching an asymptote after around 1500 cells in the simulation results, which is approximately 8–10 cm is length or just past the point where the length of the conductor is the same as its width and height.

Though the simulations presented here made simplifying assumptions about cell geometry and packing mechanics, previous research in jammed packings and conductive medium clearly provides areas for future research in optimizing the cells and packing procedures, with consequently more complex simulations. We refer the reader to several works that point to avenues for improvement. A thorough review of jammed hard-particle packing, including the effects of geometry, achievable configurations, and the lexicon for packing literature are given by Torquato and Stillinger [14]. Other literature on geometric packings also takes into account inter-particle interactions such as friction, normal forces, and gravity [15] [16]. Other relevant works on the properties of jammed packings include investigations of electrical conductivity for jammed tetrahedral particles (a theoretical treatment) [17], the conductivity of random resistor networks [18], and methods of improving contacts in jammed packing through geometry[19]. Though these researchers explored methods of improving conductivity through cell geometry and analysis through effective medium theory, our simulations are simplified by the choice of quasi-spherical cells and our analysis is done using statistical interpretation.

### Concept Implementation

The central design idea is to embed a large number of conductive cells made of a highly conductive metal in a base material matrix with very high intrinsic resistivity. These conductive cells come in direct physical contact with each other during the fabrication of the composite, and through their number and adjacency, create a connected network of contact resistances. This highly-redundant series-parallel resistor network provides a medium of charge transfer in the matrix provided the inter-element resistances – effectively the resistance at the contacts – are small.

### A. Cell Structure and Properties

**Figure 4:**
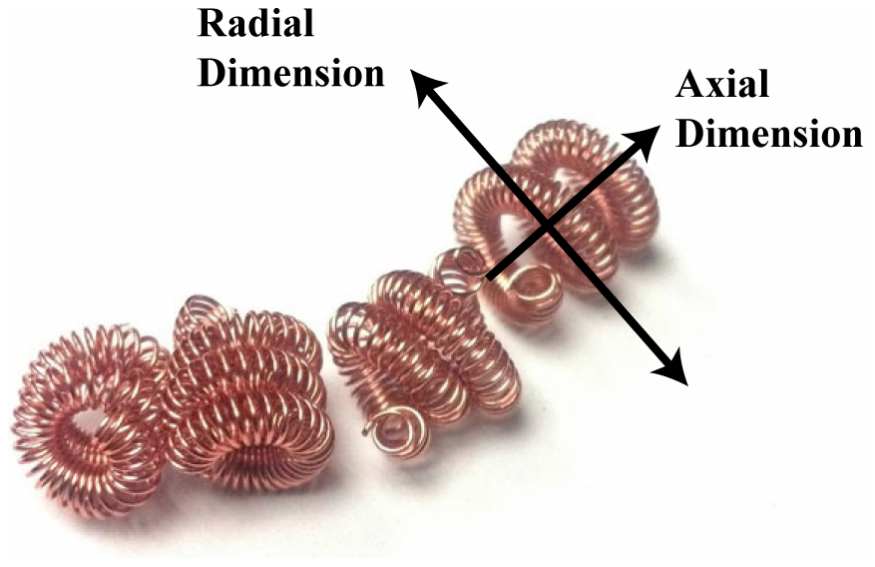
The cell design used in our tests. The labeled arrows show the dimensions of the coil referred to in the text while discussing the stiffness properties of the cells. The larger coil in the coil-of-coils design acts as the primary compliance element, able to shear in both the axial and radial dimensions. Ensembles of these cells also exhibit this property, hence making cell packings a low-stiffness internal skeleton inside the composite.

After a number of informal preliminary tests, we chose a cell design that balanced low volumetric density, low stiffness, high number of possible contacts with nearby cells, low probability of interweaving with neighbors, and ease of fabrication. The cells chosen are a ‘coil-of-coils’, in which bare copper wire (AWG 30) was wound around a 1.5 mm rod to form a thin linear coil which was then rewound around the same rod to form a 'coil-of-coils’, which was clipped at every 6 mm (approx.) (Figure 4). Although the cells have bending compliance in all directions, informal experiments show that the highest compliance exists in shear in both the axial and radial dimensions as the coil of coils slide along each other (refer to Figure 4 for labeled dimensions).

The stiffness properties of a cell packings were evaluated by applying a known strain via an Instron (Instron 5542 Universal Testing Machine, Instron Engineering Corporation, Norwood, MA) and recording the resultant force (using Instron 2530 1 kN Static Load Cell). The results showed an increase in stiffness with the incorporation of the internal cell skeleton of up to 4x compared to the pure polymer depending upon the packing fraction used for the cell packing. The various packing fractions and their relative tradeoffs in performance are discussed in following sections.

When implemented within a structure, these cells contact their neighbors throughout the network, and at each contact, the contact resistance is generally much higher than the resistance through a cell itself. Thus the network of the cells can be effectively modeled by a network of contact resistances. The contact resistance between two wires creating a circular contact patch is given by the expression



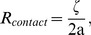
where ζ is the conductance of the terminal material and *a* is the contact patch area. This contact patch area is given by



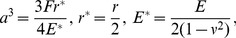
where *F* is the normal force between the conducting surfaces, *r* the radius of each cell, *E* the elastic modulus of the cell material and ν the Poisson ratio of the cell material [13].

Note that the choice of the metal to be used for these cells should have a sufficiently low Young’s Modulus to deform under contact forces and create contact patches. The larger the contact patches, the lower the corresponding contact resistance. Since the resistive properties of the composite results from the contact resistance network of the cell packings, the overall 'bulk resistivity' of a given composite sample is related to the geometry of the entire structure. Given the measured resistance R of the sample from flat plate electrodes with electrode area A and electrode separation height of L, this bulk resistivity ρ is given by



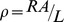
.

### B. Implementation

**Figure 5:**
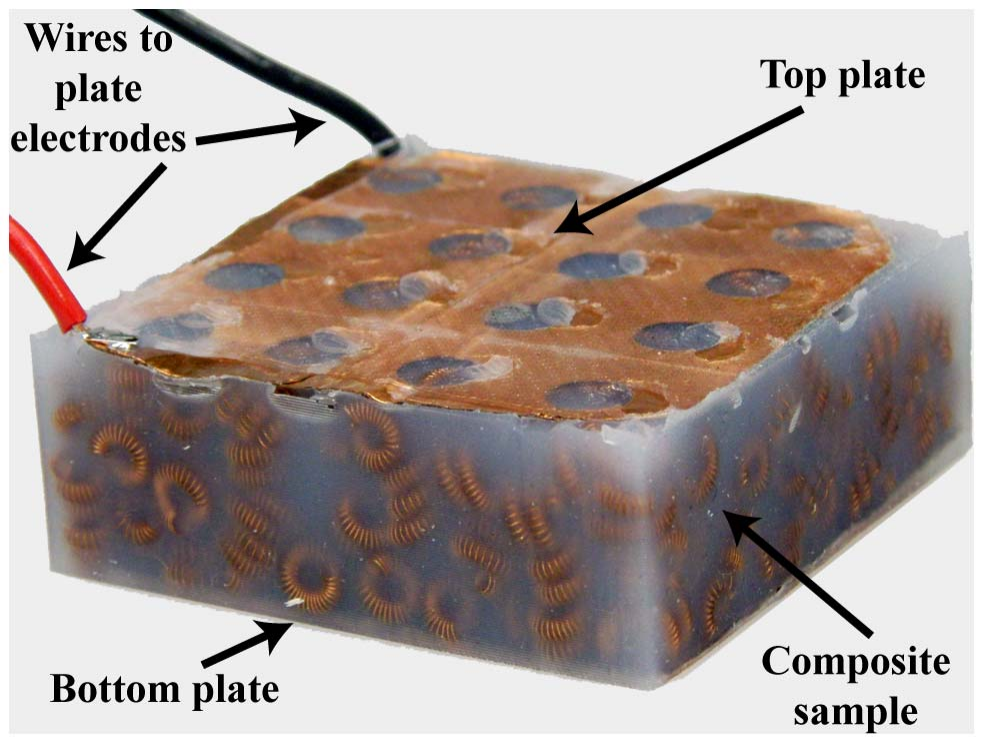
Composite sample with its parts labeled. Flat copper plate electrodes are placed on the sample packings during curing to make good electrical connections to the top and bottom faces of the composite. Since the resistive network consists primarily of contact resistances, the electrode connections need to be highly conductive in order to avoid large measurement errors.

With the central design idea calling for embedding cells directly in a matrix, we present and test three preparation methods, each with increasing conductivity but at the price of increased implementation complexity. The first and most basic, which we refer to as “unjammed packing”, simply involves randomly filling a mold with cells, giving the lowest conductivity (but still acceptable for many applications) due to low contact force (and high contact resistance) between cells and lowest mean contact number. A slightly more complex preparation, “jammed packing”, involves vibrating the mold after filling with cells in order to increase their mean number of contacts and cell-to-cell contact area, giving a higher overall conductivity. The last preparation, “pre-stressed jammed packings”, includes a load added on the cells in the mold (perpendicular to the electrode faces as in Figure 5) in order to further increase the cell-to-cell contact forces and mean number of contacts within the mold, reaching the highest overall conductivity.

A rectangular mold measuring 6 cm×6 cm is filled with conducting cells for all tests, with each cell approximately 0.6 cm in diameter (Figure 1). Once the packings are tested, a preparation of shore 10 silicone (Smooth-On, Inc., Easton, PA) is poured onto the cells to cure around the cell skeleton. Composites are tested once they have been removed from the mold.

**Unjammed packings.** This is the most basic implementation. A large number of cells are poured in the mold, in sufficient numbers to fill the entire volume of the final composite. Two flat plate electrodes are placed on the top and bottom of the cell packing. The polymer mixture is poured onto the cell packing, which is kept bulk constrained by holding a movable plastic top boundary plate touching the top electrode. Once the polymer is cured, the composite is extracted from the mold. Electrodes can be placed on the cells during curing or they can be inserted on the face of the composite post-curing. In our test cases, the average volume fraction of copper in unjammed packings was 7.4%.

**Jammed packings.** A given number of cells packed in a mold randomly are highly likely to occupy a configuration that does not have the minimum possible inter-cellular spaces. It follows from this observations that the packing fraction of random packings are not optimal and could be increased by moving individual cells strategically. In order to avoid careful relocation of individual cells into a known optimal packing configuration, an alternative method of jamming the cells is used.

Packings of cells are placed on a vibrating platform (PASCO Mechanical Driver, PASCO Scientific, Roseville, California) that oscillates vertically. The vibration profile consists of four distinct stages: 25 Hz at 6 mm amplitude for 1 minute; linearly increasing the frequency to 80 Hz over a period of 30 seconds, with amplitude linearly decreasing to 0.5 mm linearly; 80 Hz at 0.5 mm amplitude for 1 minute; and linearly reducing the amplitude of oscillation down to 0 mm over 1 minute. These stages enable the cells to explore the global phase space to find non-local minimum energy positions (fluidization), allow smooth settling of the packing, and finally, allow the attainment of the best local minima given the settled packing configuration (high frequency oscillations reduce friction between cells). This vibration profile for jamming was derived from similar work on spherical particles described in [20], [21]. Combined, the vibration profile allows the cells to occupy a quasi-jammed configuration within their container. It should be noted that since the cells are compliant, packings of cells can be “jammed” past vibration-settling, but this additional compaction deforms the cells (and is only performed for the last packing type).

The stiffness of the packings after the vibration-settling profile can be expected to increase as the cells leave very few air-gaps between neighbors. However, a significant increase in the number of inter-cellular contacts (relative to unjammed ‘gravity-settled’ packings) can also be expected since neighboring cells are likely to be in better contact with vibration-settling than in an unjammed packing. These packings have a higher volume fraction of copper, at 8.3%.

**Jammed and pre-stressed packings**. The final packing preparation further improves on the vibration-jammed packing by increasing cell-to-cell contact force, thereby lowering contact resistances. Equations for contact resistance in Section IV show that higher applied normal stress at contact points directly translate to lower contact resistances, and since applying a stress on the bulk of the packing distributes stress throughout the structure, applying a normal stress on the entire packing can be expected to reduce the measured resistance of the packing. After stepping through the vibration-packing procedure described above, a pre-stress was applied on the cell packings of 4 kPa (the choice of pre-stress magnitude is explained below in Section IV) which was maintained as the composite cures. These packings have a slightly higher volume fraction of copper at 8.8%.

### Experimental Testing

The general procedure used in the testing of cell packings and composites was twofold: evaluate the aggregate network after packing, and measure the sample resistance between top and bottom surfaces as stress is applied after compositing with a low-modulus silicone matrix (Shore 10 Silicone, Smooth-On, Inc., Easton, PA). During compositing, a boundary was placed at the top surface of the cell packing in order to prevent the cells from floating as the silicone cures around it, keeping the cell contact network generated during preparation in place.

### Uncomposited Packings

Cells of approximately 6 mm in diameter are prepared in a rectangular 6 cm×6 cm mold and a flat copper plate electrode is placed on the top and bottom face of the packing (Figure 5). The resistance of the sample is measured using a standard 4-wire setup, passing 100 mA of current through the sample and measuring the potential difference across. In order to evaluate the load-dependency of the structures, which can be significant due to the decreases in contact resistance with applied force, this setup is placed on an Instron machine (Instron Model 5542), which applies a stress on the top surface of the packing through a square indenter plate (6 cm×6 cm).

**Figure 6:**
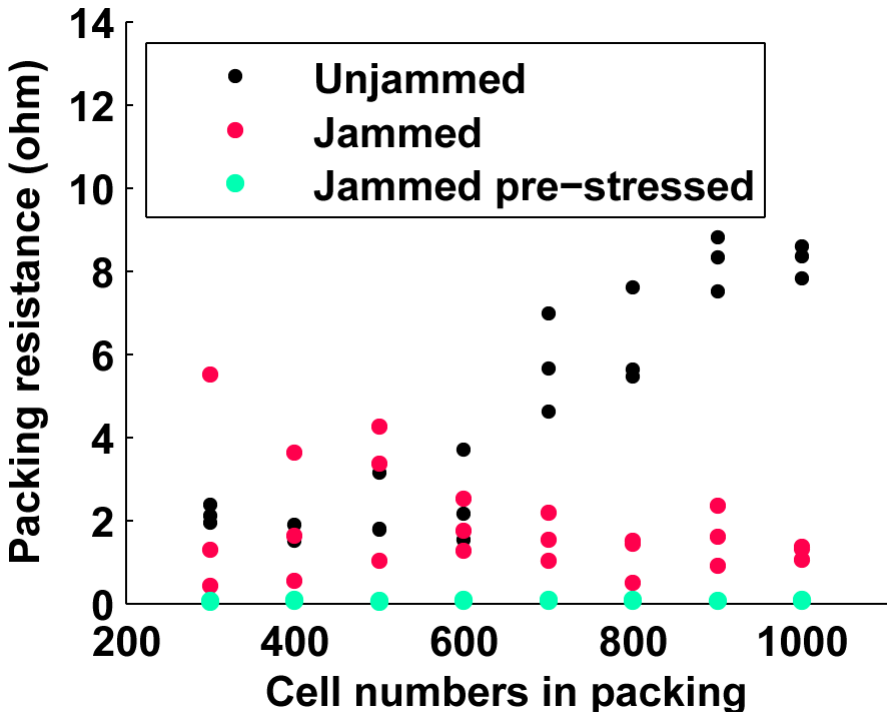
Resistance vs. cell numbers for all three types of cell packings. Unjammed packings clearly show the increase in packing resistance with cell count. The increase in resistance with packing size in jammed packings and jammed, pre-stressed packings is relatively low and somewhat masked by high variance between trials. Jammed, pre-stressed packings have extremely low resistance for all cell counts, although the resistance increases with cell count for all three types. Refer to subsequent plots detailing fits on these plots and evaluating the linear trends.

For each type of packing, samples are made containing increasing numbers of cells, from 300 to 1000 cells in steps of 100 cells. At each of these eight cell counts (which correspond to an increased height of the packing), three different samples are made to capture some of the variability that results from the random nature of the cell configurations (which should be low for these sample sizes, as predicted by the simulation results described in section II). Figure 6 shows the resistance of the samples as a function of sample size (numbers of cells) for the three preparations.

**Figure 7:**
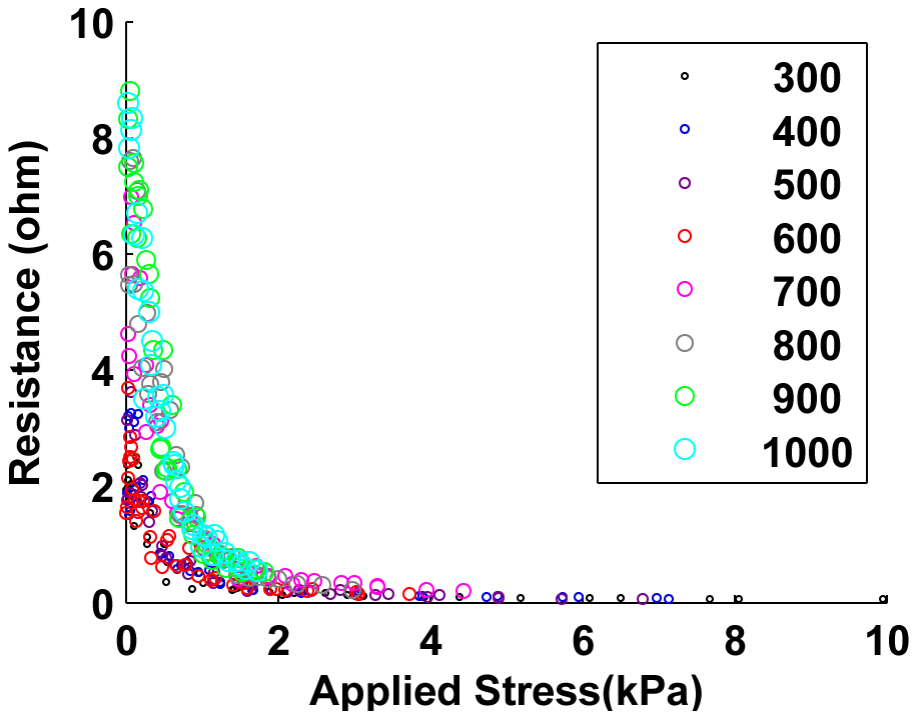
Performance of unjammed cell packings. Increasing stress decreased the measured resistance of all cell packings; the greatest change was at low applied stresses.

**Figure 8:**
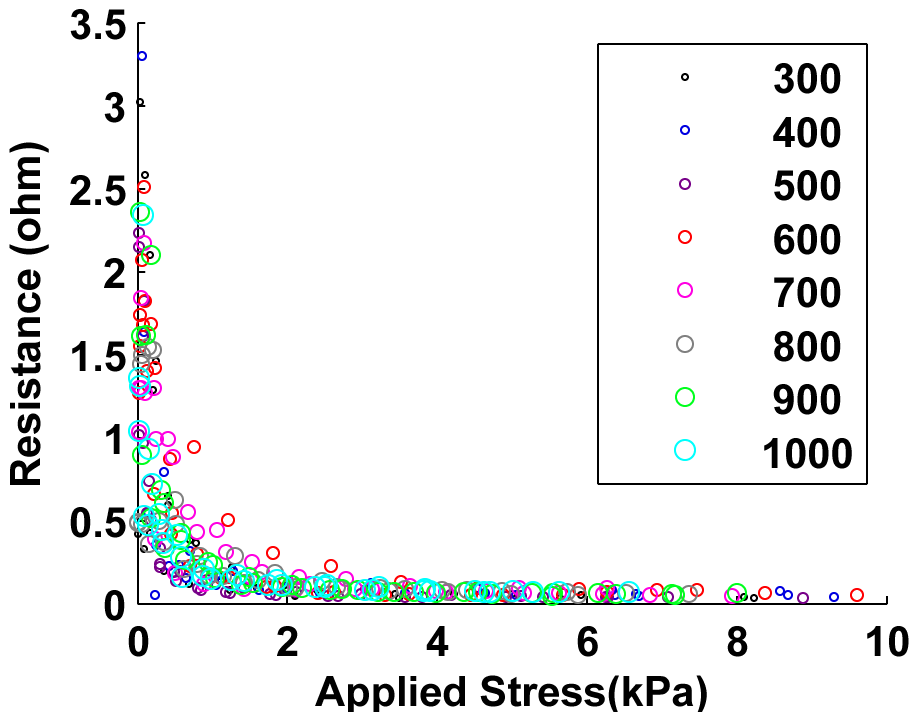
Performance of jammed cell packings. Similar to Type 1 packing, increasing stress showed decreased measured resistance of all cell packings.

**Figure 9:**
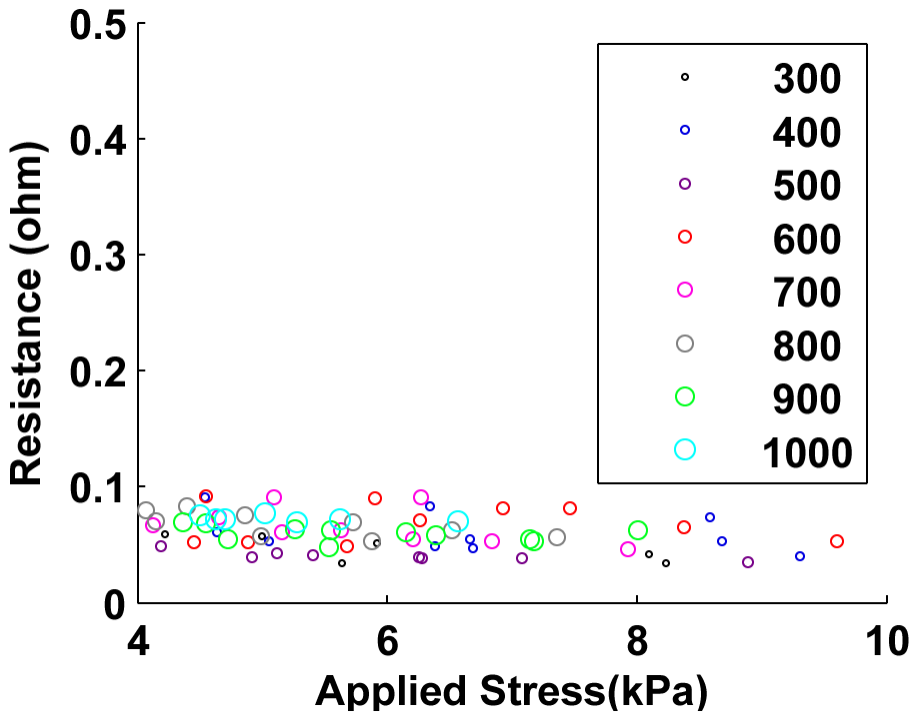
Jammed and pre-stressed cell packings. The results are identical to jammed packings with data shown from 4

In order to demonstrate the effect of applied load on the sample resistance, we applied compressive forces to the samples via the Instron machine. Figure 7, Figure 8 and Figure 9 show the results from 24 sample preparations as the stress is applied for the “unjammed” and “jammed” packings, respectively. “Pre-stressed jammed” packings are, by definition, “jammed” packings after 4 kPa of stress has been applied, which is the equivalent of the “tail” of the results for jammed packings.

### Composited Samples

**Figure 10:**
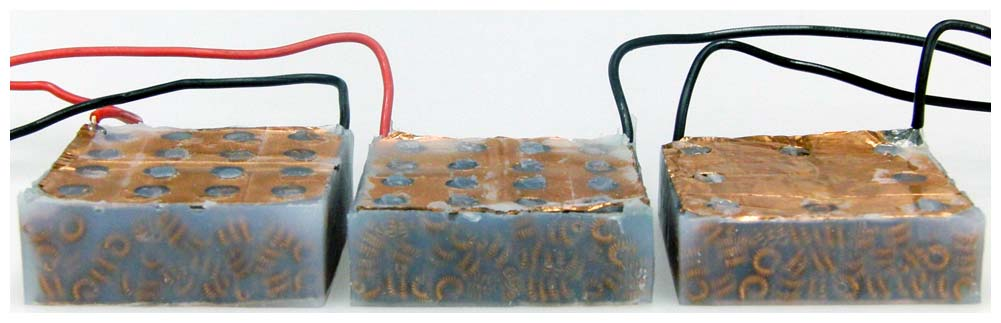
Image of a 300 cell sample of each of the three types of composite. Unjammed: left, Jammed: middle, Jammed, pre-stressed: right. Notice the decreasing height of the samples as the packing fraction increases from Unjammed to Jammed pre-stressed.

**Figure 11:**
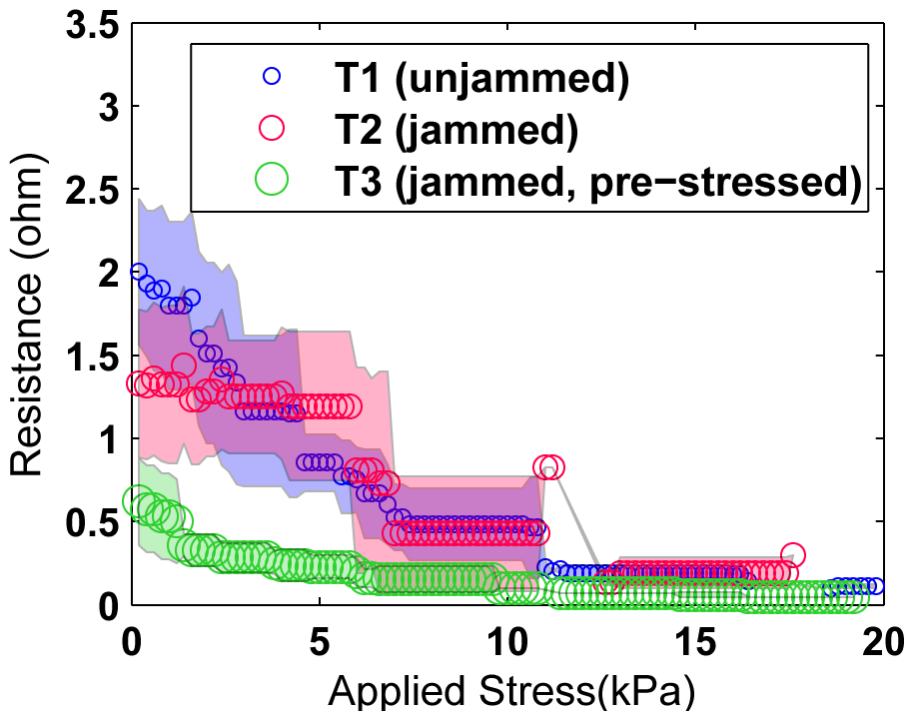
Resistance (mean of trials) vs. Applied Stress on the three types of composites. The decrease in resistance is less steep in composites that in corresponding packings. Jammed, pre-stressed composite is the most insensitive to applied stress since its performance corresponds to the tail end (low variance) of a jammed packing.

**Figure 12:**
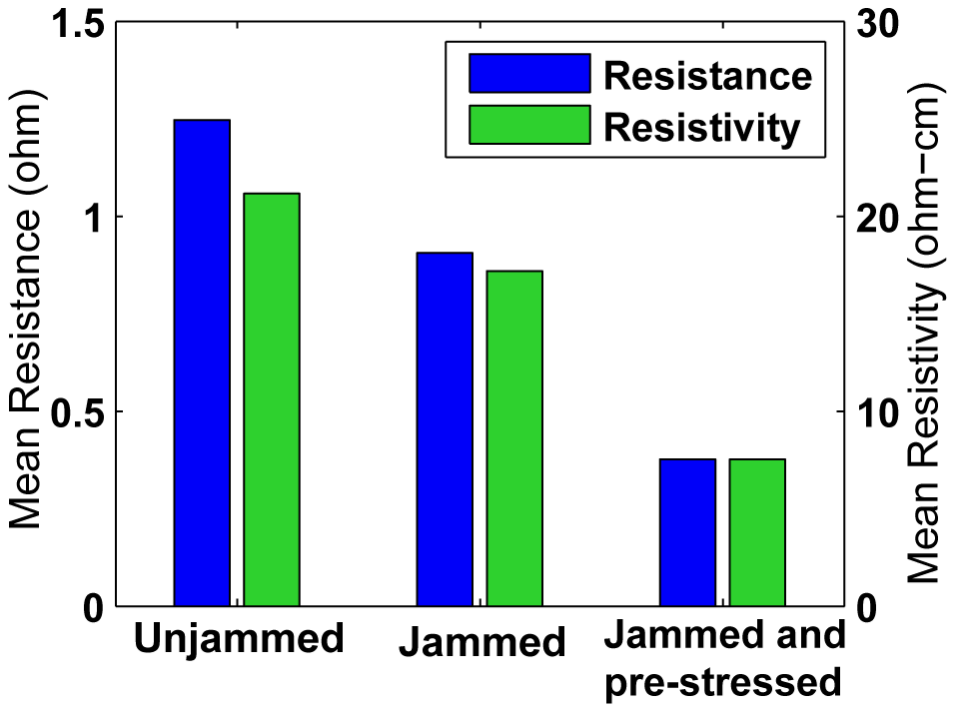
Mean resistance (left bars) and resistivity (right bars) for the three types of composite. The measured resistances of Figure 11 are averaged to obtain the mean resistance, which is used to compute the resistivity. Note the resistivity of composite samples, while low compared to silicone rubber, is higher than the resistivity of the packings embedded in them. We believe that the polymer’s cure-time wetting effects might be responsible for this increase. Refer to the text for a detailed discussion.

After testing a fairly large number of uncomposited packings to examine the effects of preparation type and applied load on the resistance performance of the preparation, we test a smaller number of composited samples (with shore 10 silicone (Smooth-On, Inc., Easton, PA)) made from these packings. A sample of each of the three preparation types were fabricated, containing 300 cells each (Figure 10). The curing time is 24 hours, at which point the samples are extracted from the mold and any additional silicone trimmed away. The samples contain two electrodes embedded on the top and bottom that are kept in contact with the cells closest to them during curing by bulk-constraining the tested packings. Composites of the third type, “Jammed with pre-stress”, are cured while a known mass (calculated to apply 4 kPa of stress on the cell network) is placed on top of the packing. The choice of this stress value was made after observing a plateauing effect in resistivity of jammed packings at applied stresses greater than 4 kPa (Figure 9). After loading in the same manner as the uncomposited packings (described above), the performance of the composites (resistance vs. stress) is shown in Figure 11. A bar graph of the resistance and resistivity for the composite samples are shown in Figure 12. Since the composited structures are considerably stiffer than the uncomposited packings, the variation in resistance of the composites with added stress is much lower than the corresponding packing.

**Figure 13:**
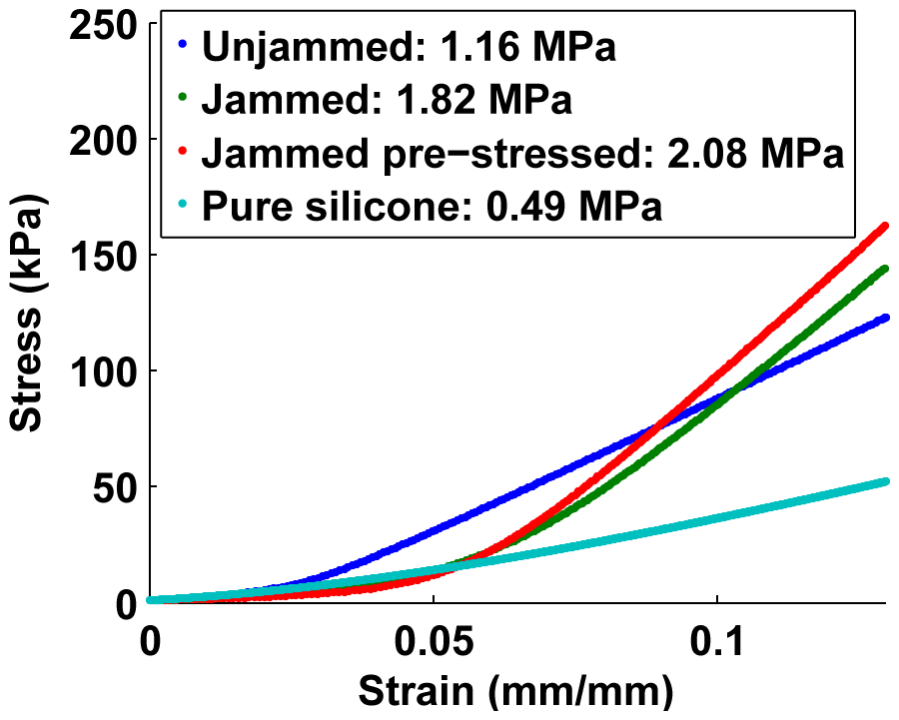
Stiffness of the three composite types compared to pure silicone rubber.

**Figure 14:**
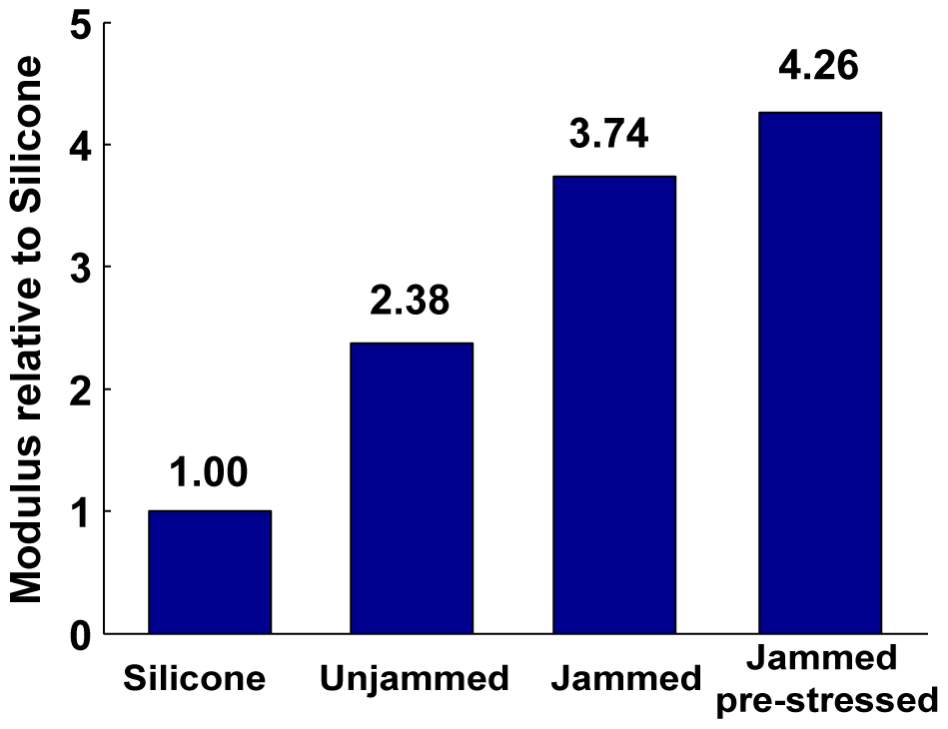
Young’s Modulus of the three composite types compared to pure silicone rubber.

To obtain the modulus of the composite samples, they are placed on the Instron for a stress-strain test. The Instron applies increasing amounts of strain on the composite samples (up to 3 mm of travel) and measures the registered stress on a load cell. All tests are preceded by 5 cycles of pre-stress and release, ensuring that contact is well-established between the face-electrodes and the surface cell layers in the sample. The plot of stress with strain for the three types of composite is compared with that of pure silicone rubber of the same dimensions (and the same Shore hardness) (Figure 13). Each composite sample shows a smooth increase in stiffness to a steady value as the entire bulk of the composite begins to compress. The behavior of each sample at 0.8 mm/mm strain is used to calculate the Young’s Modulus for each sample, shown in the bar chart in Figure 14.

These results show that composites with unjammed aggregate are about 2.25 times as stiff as pure silicone. While the best resistance performance is provided by “jammed and pre-stressed” packings (Figure 12), the stiffness increase is about four fold. This stiffness factor increase is a function of the modulus of the matrix material, however stiffer materials will show a smaller relative increase in stiffness. Depending on the application in hand, the appropriate tradeoff of electrical performance and stiffness can be made.

### Measurement Error

The resistance measurements reported in this paper were taken by a simple 4-wire setup where a current supply (GPS-3303, Good Will Instrument Co. Ltd., Taiwan) supplied 100.0 mA of current through the sample (packing or composite) and a voltmeter (Fluke 117, Fluke Corporation, Everett, Washington, USA) measured the voltage across the sample alone (in mV). The resistance was obtained by dividing the voltage across the sample by the constant current through the sample. The resolution of the constant current source is ≤ 0.2%+3 mA, which gives an error of 3.2 mA for a 100 mA mean current. The error in voltage measured comes from the voltmeter, which is rated for ±0.5% of reading. These values give a mean error of 3.48% for the resistance measurements across all samples, and a maximum error of 1 m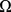 (on the smallest recorded resistance of 0.029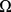, measured on a jammed and pre-stressed packing).

## Discussion

The results presented above suggest several conclusions regarding the performance of the cell packings as well as the composites. Analysis of these observations, as well as that of errors in measurements and estimation is presented below.

### Resistance changes with stress and sample size

Increasing applied stress on the samples increases the intercellular contact forces, which drives down the contact resistance and therefore the bulk resistance of the entire sample. As in our simulations, this effect of applied bulk stress on resistance decreases as stress increases, since the rate of increase of the contact resistance decreases as contact forces rise (refer to equations for contact resistance in Section IV). Since the polymer material increases the stiffness of the sample as well as inhibits free compression of cells with their neighbors, the sensitivity of applied bulk stress on resistance is stronger in cell packings than in the corresponding composite samples (compare Figure 7, Figure 8 and Figure 9 with Figure 11).

**Figure 15:**
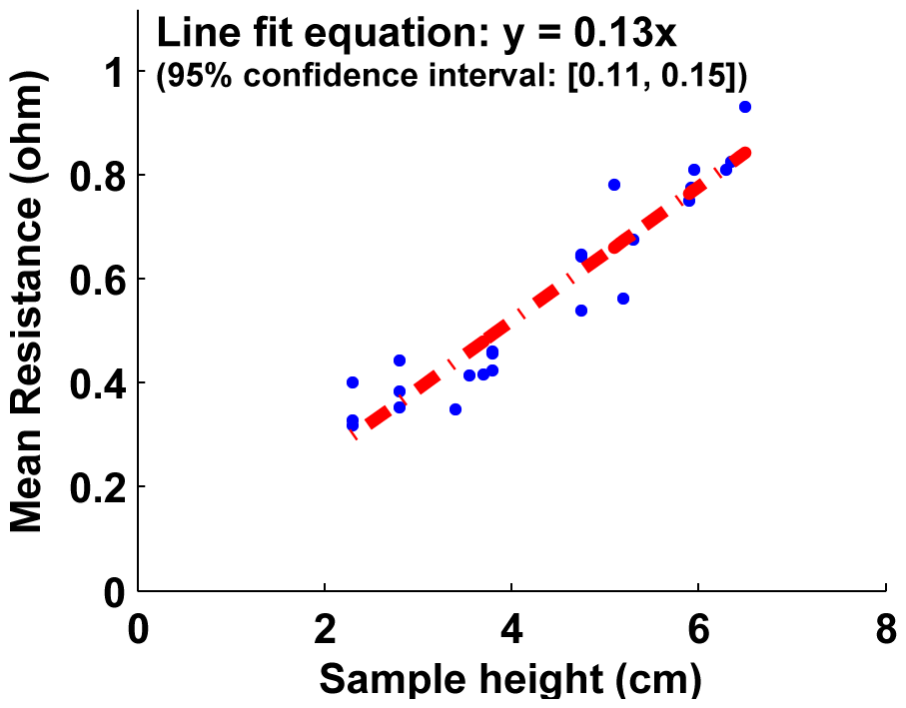
Mean resistance as a function of number of cells for unjammed cell packings. The linear fit with a positive slope (entire 95% confidence interval is positive) shows the resistance of unjammed packings increase with increasing sample height. The slope of this R vs. L plot is proportional to the effective bulk resistivity of the packing. Using the geometry of the sample, this provides a method to calculate the resistivity of unjammed packings: 4.7±0.34 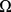-cm.

**Figure 16:**
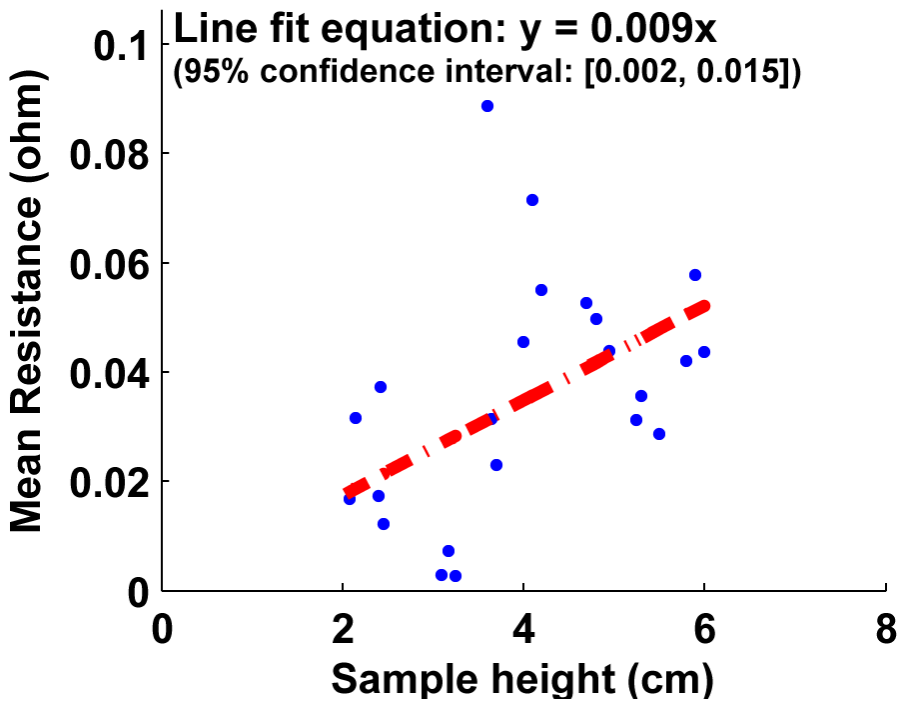
Mean resistance as a function of number of cells for jammed cell packings. Similar to unjammed packings, the linear fit for jammed packings has a positive slope and the slope of the R vs. L plot is proportional to the effective bulk resistivity of the packing. Using the geometry of the sample, the resistivity of jammed packings is computed to be 0.31±0.12 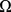-cm.

**Figure 17:**
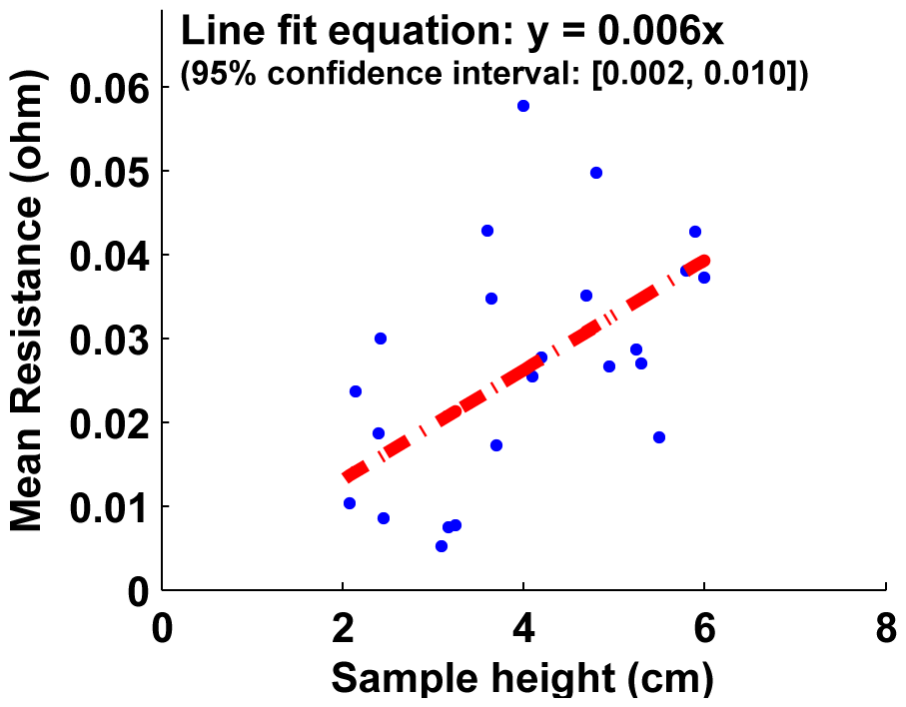
Mean resistance as a function of number of cells for jammed, pre-stressed cell packings. Similar to jammed packings, the linear fit for jammed, pre-stressed packings has positive slope (95% confidence) and the slope of the R vs. L plot is proportional to the effective bulk resistivity of the packing. From the plot, the resistivity of pre-stressed jammed packings is 0.23±0.074 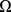-cm.



The resistance of samples with increasing height (larger cell numbers) is shown on Figure 15, Figure 16 and Figure 17. For both unjammed and jammed packings, linear fit through the data highlights the trend observed. The 95% confidence interval for the slopes of all three line fits are positive, and hence we can infer a positive linear increase of resistance exists with sample height. This linear increase of resistance with height is consistent with an ohmic conductor whose resistivity is given by

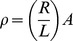
where *R*  =  resistance from top to bottom plate, *ρ*  =  resistivity, *L*  =  height of the sample and *A*  =  cross-sectional area of the faces used as electrodes.

The slope of the linear fit of resistance-length plots is thus proportional to the resistivity (with the constant cross-sectional area as the proportionality constant). Using the linear fit slopes of unjammed and jammed packings (and the confidence interval of these slopes), the resistivity is obtained to be **ρ  =  4.7**±**0.34 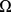-cm for unjammed packings** and **ρ  =  0.31**±**0.12 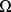-cm for jammed packings**. Pre-stressed jammed packings are essentially jammed packings observed at stresses past the ‘steady-state cutoff’, and thus the portion of the plot above the stress cutoff for jammed packings on Figure 8 is shown on Figure 9 as the resistance-stress property of pre-stressed jammed packings. The resistivity for pre-stressed packings, obtained as before for unjammed and jammed packings, is **ρ  =  0.23**±**0.074 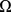-cm**.

### Performance of packings and composite

Figure 7 and Figure 8 provides the immediate observation that while the resistance for jammed packings range below 3.5 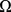 while for unjammed packings this range is below 9 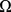. This shows that jamming a cell packing reduces the resistance of the packing. Such a trend is also seen in the performance of the composited samples (Figure 12).

Figure 11 shows the effect of applied stress on the composite samples. At low stresses, the variability of measured resistance is high and at higher stresses the cells are compressed together in the composite, increasing the contact forces between neighbors and reducing resistance. Pre-stressed jammed composite trials show the least variability (and resistance) since they constitute the highest packing fractions and the most stressed embedding.

Although the effect of jamming the cell packings is similar in cell packings and corresponding composites, the composites have significantly higher empirical resistance value than the packings which is embedded in it, as seen from Figure 12. To account for this, we hypothesize that the polymer, as it cures, wets the contacts between the copper cells and reduces the contact forces and/or area, thus increasing the contact resistances between two adjacent cells. In special cases where contacts between cells in the packing are weak, the presence of the polymer may even break contacts between adjacent weakly connected cells entirely, thus altering the connectivity network. It is reasonable to assume that these alterations of network connectivity are much more likely in unjammed packings than jammed packings, since unjammed cells are only weakly connected under gravity and there exist many possible alterations to the packing configuration that can be made by the curing polymer.

## Conclusions and Future Work

The central idea of this paper, namely the formation of conductive composite via direct embedding of conductive cells, is shown to be a potential alternative to existing methods based on intrinsically conductive materials and microscopic aggregate additives. The cellular approach discussed herein has the distinct advantage of having very little dependence on the composite matrix material. We have shown that the conductivity of the aggregate packing is dependent on the preparation of the sample in terms of the density of the packing and the force applied. Direct cell embedding is largely independent of the specific geometry of the structure or the intrinsic properties of the matrix material, and hence can be used for a wide range of applications. In this paper we described three different potential implementation methods of direct cell embedding, each trading off stiffness of the resultant composite for low resistivity in different amounts. Several customizable aspects exist in this design, such as the structure of the embedded cells, the number of cells, the choice of composite matrix (a polymer or more rigid material) and the geometry of the mold in which the composite is cast. We believe that the presented method of directly embedding low density conductive cells to arrive at a contact-connected aggregate for conductive composites, has the potential of providing a new manufacturing method of conductive materials with fewer downsides than existing methods.

While we were able to successfully demonstrate composite structures with very low resistivity, using a process that is easy to implement and can be applied to a wide range of materials and geometries, there are a number of limitations to the work presented here that we would like to address in the future. While we conducted informal tests on a few other “cell” designs, including linear coils, future work could involve a more thorough investigation of cell designs to provide even better conductivity and lower volume fractions of aggregate material. Additionally, the size of the cells should be further investigated, including the fabrication of micro- and nano-scale cells for conductive structures with much smaller size features, such as thin films. However, the general concept should be applicable across a wide range of size scales. Finally, in order to give the lowest resistance structures without the need for pre-loading, methods to increase the contact area or decrease contact resistance between cells might be investigated, such as through wetting with a conductive liquid or a solder-like tinning of the cells which are heated after packing to join them at the contacts.
